# Identification of small molecules for accelerating the differentiation of GABA interneurons from human pluripotent stem cells

**DOI:** 10.1093/jmcb/mjaa002

**Published:** 2020-01-26

**Authors:** Luping Shen, Fang Yuan, Yuan Hong, Min Xu, Yao Hu, Yan Liu

**Affiliations:** 1 Institute for Stem Cell and Neural Regeneration, Key Laboratory of Targeted Intervention of Cardiovascular Disease, School of Pharmacy, Nanjing Medical University, Nanjing 211166, China; 2 State Key Laboratory of Reproductive Medicine, Nanjing Medical University, Nanjing 211166, China; 3 Co-innovation Center of Neuroregeneration, Nantong University, Nantong 226019, China

**Dear Editor,**

Here, we present a method to accelerate the differentiation of γ-aminobutyric acid (GABA) interneurons (GINs) from human pluripotent stem cells (hPSCs). Normal brain function requires balanced levels of excitation and inhibition of neurotransmitter. GINs are the main inhibitory neurons in the central nervous system, and they are thought to play a critical role in sculpting inhibitory network dynamics in the cerebral cortex (Rossignol, 2011). Thus, dysfunction of GINs induces many neurological diseases, such as epilepsy, major depression, anxiety, and autism. Since it is difficult to recover from the dysfunction/loss of GINs in the neural network, exogenous GIN transplantation has been considered a potential therapeutic strategy for the treatment of GIN-associated neurological diseases. Although reported methods offer various ways to differentiate high-purity human GINs from hPSCs, it still takes >1 month to obtain a high proportion of GINs (Table 1). Moreover, the protracted timing of human GIN specification and subtype maturation remains a key challenge that hampers the routine application of hPSC-derived lineages in disease modeling and regenerative medicine studies. Additionally, the current differentiation method mainly relies on co-culture with rodent cortical neurons/astrocytes and cell sorting, which limits the application of human GINs for cell transplantation and therapy. Therefore, it is urgent to establish a time- and cost-effective, highly efficient non-xenogenic differentiation system for differentiating human GINs. Here, we sought to identify small molecule (SM)-based conditions that accelerate the differentiation of human GINs without adding any components with heterologous origins or performing genetic manipulations.

To accelerate the differentiation progress to <30 days, we examined the ventral patterning efficiency of Smoothened agonist (SAG) instead of purmorphamine (PUR) (Supplementary Figure S1A), since SAG was more efficient and safer than PUR, as previous reported (Wang et al., 2010). Following the application of the embryoid body (EB) protocol for neural induction (Figure 1A and B), PUR (1.5 μM) or SAG (0.1 and 1.0 μM) was given from Day 10 to Day 25 (Liu et al., 2013). To compare the expression of the anterior ventral transcription factors, we performed quantitative polymerase chain reaction (qPCR) on Day 17. The qPCR results showed that compared with PUR treatments, both 0.1 and 1.0 μM SAG can enhance the expression of ventral transcription factors *NKX2.1*, *LHX6*, *LHX8*, and *ISLET1*. *PAX6*, a dorsal transcription factor, was significantly decreased in the SAG 1.0 μM group than in the PUR group (Figures 1C; Supplementary Figure S1B). Strikingly, we found that the messenger RNA (mRNA) level of *NKX2.1* was 100 times higher in the SAG 1.0 μM group compared with that in the PUR group (Figure 1C). To further confirm the results, we characterized the identities of neurons on Day 30 by immunostaining (Figure 1D). Both SAG 1.0 μM group and PUR 1.5 μM group yielded a high percentage of NKX2.1-positive cells (SAG 1.0 μM, 95.7%; PUR 1.5 μM, 90.3%). Furthermore, we tested whether 1.0 μM SAG and 1.5 μM PUR treatment had similar effects on the differentiation of GINs. A high percentage of GABA-positive (GABA^+^) neurons were generated in both PUR 1.5 μM group and SAG 1.0 μM group (PUR 1.5 μM, 91.9%; SAG 0.1 μM, 77.7%; SAG 1.0 μM, 94.4%). Moreover, neuronal differentiation was not affected by 1.0 μM SAG, according to the percentage of TUJ1-positive or MAP2-positive cells (Supplementary Figure S1C). Taken together, compared with 1.5 μM PUR, treatment with 1.0 μM SAG showed a significantly higher efficiency of ventralization, and a slightly higher GIN differentiation rate. Moreover, water-soluble reagents (e.g. SAG) usually show less toxicity than DMSO-soluble reagents (e.g. PUR). We therefore used SAG as a sonic hedgehog (SHH) pathway activator instead of PUR.

**Figure 1:**
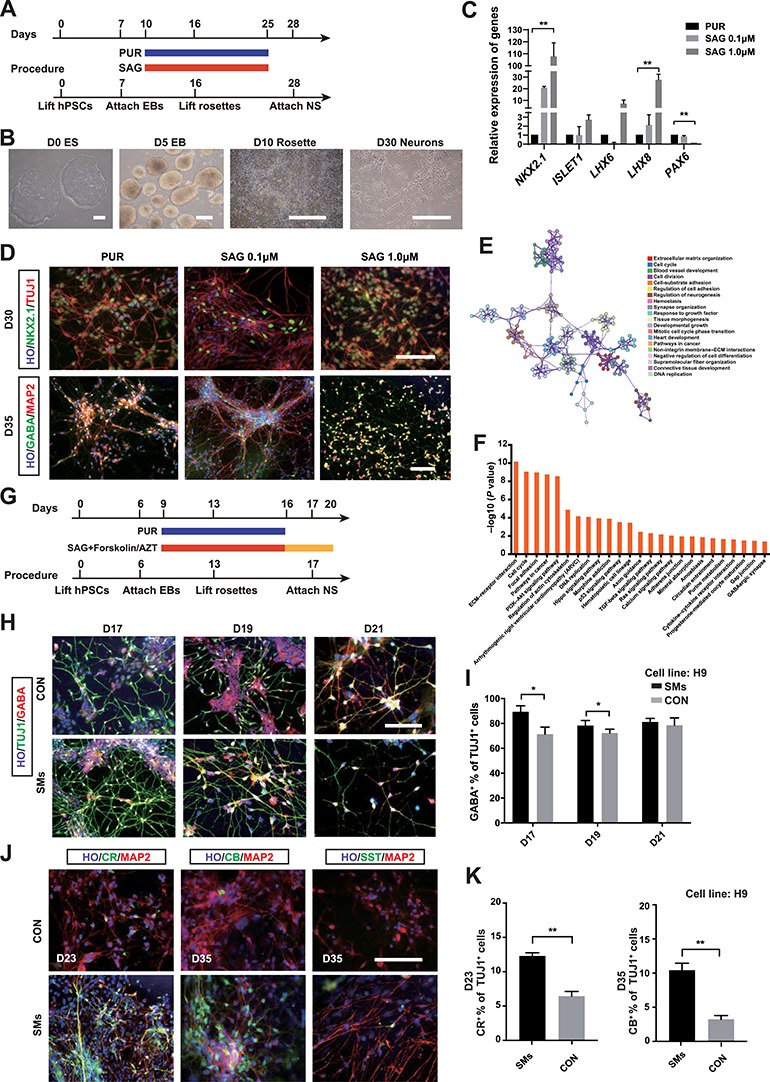
Optimization of conditions for accelerating the generation of GINs from hPSCs. (**A**) Timeline of GIN generation. (**B**) Illustration of differentiation of GINs from hPSCs. Scale bar, 100 μm. (**C**) Fold change of mRNA expression levels in different groups of H9 cells at Day 17. The data are presented as mean ± SEM. *n* = 3; **P < 0.01. (**D**) Representative images of NKX2.1 and TUJ1 immunostaining. Scale bar, 100 μm. (**E**) Network of enriched GO terms, colored by cluster ID, where nodes that share the same cluster ID are typically close to each other. (**F**) KEGG pathway analysis of upregulated and downregulated genes in the SMs group. (**G**) Schematic diagram showing the timing of administration of SAG, Forskolin, and AZT during the differentiation. (**H**) Immunostaining for GABA^+^ neurons, expressing GABA (red) among the neurons (TUJ1), from D17 to D21. Scale bar, 100 μm. Cell line, H9. (**I**) Percentage of the total cells expressing GABA during the differentiation. More than 5000 cells from random fields were manually counted in each condition. The data are presented as mean ± SEM. *n* = 3; *P < 0.05. (**J**) Immunostaining for GIN subtype markers CR, CB, and SST in H9 cells. Scale bar, 100 μm. (**K**) Percentage of the total cells expressing CR and CB during the differentiation. More than 1500 cells from random fields were manually counted in each condition. The data are presented as mean ± SEM. *n* = 3; **P < 0.01.

**Table 1:** Summary of reported differentiation methods.

**Conversion/differentiation**	**Patterning factors**	**Converted factors**	**Cell sorting**	**Co-culture**	**GINs**	**Time (days)**	**References**
hPSC–forebrain interneurons	SB431542, BMPRIA, Y27632, DKK1, purmorphamine	N/A	Yes	Yes	75.8% ± 2.3%	35	Nicholas et al., 2013
hPSC–forebrain interneurons	LDN193189, SB431542, SAG, IWP2, FGF8, GDNF, BDNF, DAPT	N/A	Yes	No	84.4% ± 3.4%	60	Kim et al., 2014
hPSC–forebrain interneurons	LDN193189, SB431542, IWP2, SAG, FGF8, GDNF, BDNF, DAPT	N/A	Yes	No	88.8% ± 2.1%	30	Cunningham et al., 2014
hPSC–GINs	N/A	ASCL1, DLX2, LHX6, miR9/9*-124	No	Yes	84.5% ± 3.5%	42–50	Sun et al., 2016
hPSC–GINs	N/A	ASCL1, MYT1L, DLX2	No	Yes	89.1% ± 3.5%	35	Yang et al., 2017
hPSC–GINs	SAG, Forskolin, AZT	N/A	No	No	89.3%	14	

SB431542, selective TGF-β1 receptor ALK5 inhibitor; DKK1, Dickkopf-related protein 1; IWP2, inhibitor of WNT production-2; FGF8, fibroblast growth factor 8; BDNF, brain-derived neurotrophic factor; GDNF, glial cell derived neurotrophic factor; ASCL1, achaete-scute complex-like 1; DLX2, distal-less homeobox 2; LHX6, LIM/homeobox protein 6; MYT1L, myelin transcription factor 1-like.

As SAG alone is not able to accelerate the differentiation process, we explored which SMs could shorten the time of GIN generation. We utilized original microarray datasets GSE83896 (Pfisterer et al., 2016) from the NCBI-Gene database (available online: https://www.ncbi.nlm.nih.gov/geo). These data sets are from a time course microarray experiment that identifies transcriptional changes in response to the exposure of human fibroblasts to a different combination of SMs during direct neuronal reprogramming. This study suggested that Forskolin enhanced neuronal reprogramming efficiency (Pfisterer et al., 2016). Therefore, we exported the Forskolin-related data from this database, and the differentially expressed genes (DEGs) were sorted by using the Database for Annotation, Visualization and Integrated Discovery (DAVID) program to perform gene ontology (GO) enrichment analysis and Kyoto Encyclopedia of Genes and Genomes (KEGG) pathway analysis.

GO analysis showed that Forskolin could change many important activities, including nervous system development and the generation of neurons. Then we listed the top 20 terms that were significantly influenced by Forskolin (Figure 1E; Supplementary Table S1). As shown in the KEGG pathway enrichment analysis (Figure 1F), GABAergic synapse pathways were significantly increased in the Forskolin group, together with the other related pathways listed in Supplementary Table S2. The bioinformatics results suggested that Forskolin may promote the differentiation of GINs.

Inspired by our previous study that azidothymidine (AZT) effectively promotes the differentiation and enhances the maturation of hPSC-derived neurons (Hu et al., 2018), we hypothesized that AZT could accelerate the differentiation of GINs. Thus, we modified the protocol of GIN differentiation by using the combination of SAG (1.0 μM), Forskolin (10 μM) and AZT (10 μM) (Figure 1G). Human embryonic stem cells (H9) and induced PSCs (IMR90-4) were treated with 1.0 μM SAG and 10 μM Forskolin from Day 9 to Day 16 and 100 μM AZT from Day 16 to Day 20. Intriguingly, the combination of SMs significantly accelerated the differentiation of hPSC-derived GINs. For the H9 cell line, a small population of GINs were observed at Day 14 in the no-treatment group, but >15% GINs were found in the SMs group (Supplementary Figure S1D and E). At Day 17, the SMs increased the percentage of GABA^+^ neurons (the percentage of GABA^+^ cells in TUJ1^+^ cells) from 71.4% (CON) to 89.3% (SMs), whereas the percentage of TUJ1^+^ neurons was not affected (Supplementary Figure S1F). At Day 19, a significant difference was observed between the two groups (SMs: 78.3%; CON: 72.3%). However, after withdrawing the treatment of AZT at Day 20, there was no significant difference at Day 21 (Figure 1H and I). For the IMR90-4 cell line, the SMs also significantly accelerated differentiation (Supplementary Figure S1G). Our results suggested that the combination of SMs enhanced the generation of GINs as early as Day 14. Additionally, to determine whether the combination of SMs could promote GIN subtype differentiation, we identified GIN subtypes by immunostaining for calretinin (CR), calbindin (CB), and somatostatin (SST). Compared with the control group, the percentage of CR^+^ cells was increased from 6.44% (CON) to 12.3% (SMs) at Day 23 (Figure 1K), and the percentage of CB^+^ neurons in the SMs group was significantly increased compared with the control group (Figure 1K, SMs: 10.74%; CON: 3.61%) at Day 35. In short, SMs accelerated the differentiation of GIN subtypes in both CR and CB subtypes. Because of the late generation of SST, the SST^+^ cells were barely observed by immunostaining before Day 35 in previous studies. Following our modified protocol, a group of SST^+^ neurons was generated in the SMs group, whereas none was observed in the control group (Figure 1J). Taken together, the results indicated that this combination of SMs could accelerate the generation of human GIN subtypes.

Here, we present a protocol to rapidly generate GINs from hPSCs as early as Day 14. This protocol significantly shortens the time cost for differentiating GINs (Table 1). It offers a safe approach for obtaining high purity GINs without genetic modifications, which could be applied for neural disease modeling or mechanism exploring studies.

*[Supplementary material is available at Journal of Molecular Cell Biology online. This study was supported by the Strategic Priority Research Program of the Chinese Academy of Sciences (XDA16010306), the National Natural Science Foundation of China (81922022, 91849117, and 81471301), the National Key Research and Development Program of China (2016YFC1306703), the Jiangsu Outstanding Young Investigator Program (BK20160044), and the Jiangsu Provincial Innovation Program, Postgraduate Research & Practice Innovation Program of Jiangsu Province.]*

## Supplementary Material

JMCB-2019-0322_R3_Finalized_Supplementary_File_mjaa002
