# Supplementary material for: Identification of small molecules for accelerating the differentiation of GABA interneurons from human pluripotent stem cells
Source: J Mol Cell Biol. 2020 Jan 26;12(3):245–8. doi: 10.1093/jmcb/mjaa002 (PMC7181716; doi:10.1093/jmcb/mjaa002)
Supplement: JMCB-2019-0322_R3_Finalized_Supplementary_File_mjaa002 [file jmcb-2019-0322_r3_finalized_supplementary_file_mjaa002.pdf]

Supplementary Figure

Figure S1

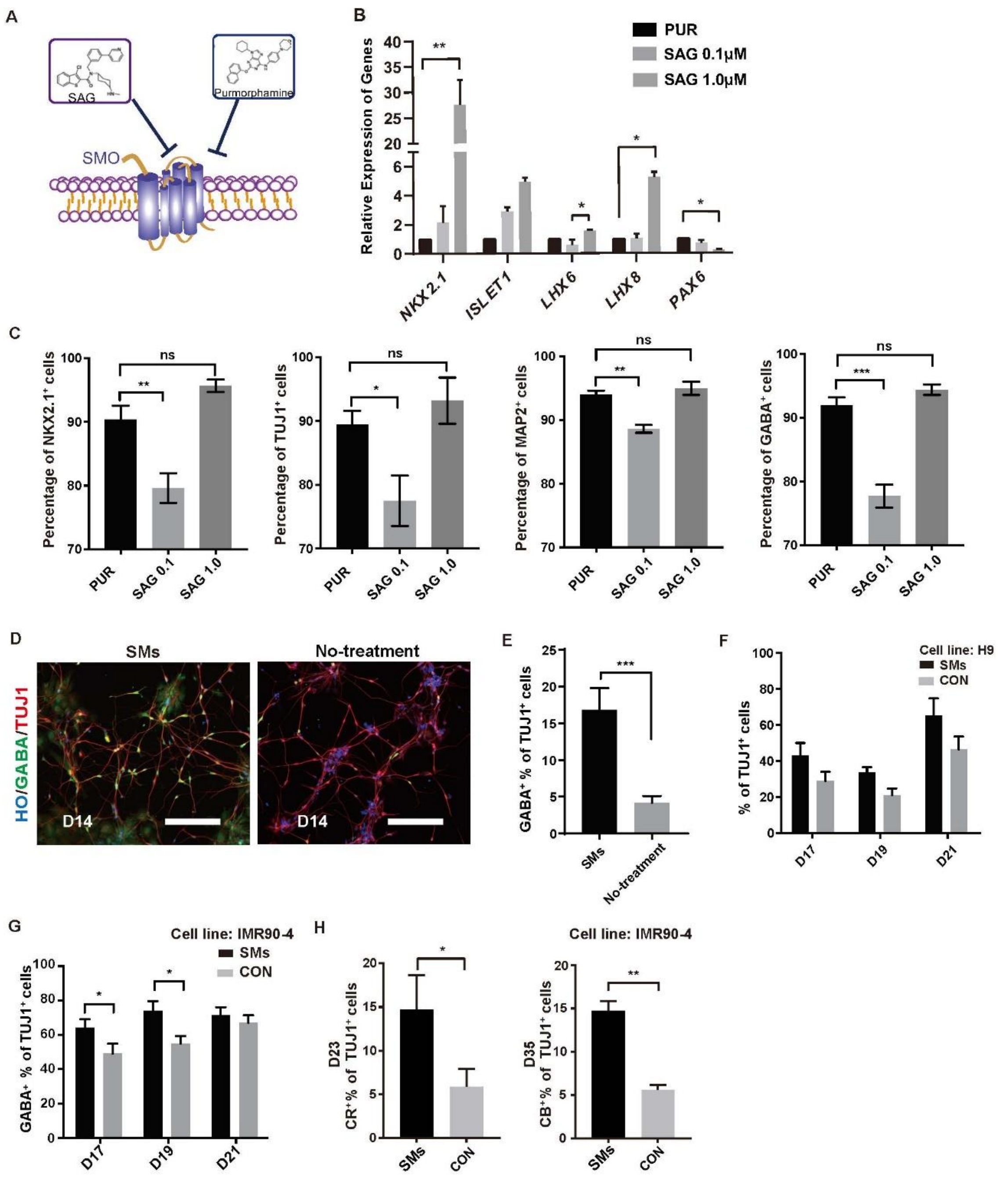

Figure S1. Optimization of conditions for efficient generation of GINs.

A. Schematic view of the molecular structure of SAG and purmorphamine.
B. The mRNA expression levels for derived neurospheres. Cell line, IMR90-4. n=3
C. Percentage of the total cells expressing NKX2.1, TUJ1, GABA and MAP2 during differentiation. n=3. *, p<0.05; **, p<0.01; ***, p<0.001. *Error bars* represent S.E. Cell line, H9.
D. Immunostaining of the GABA interneurons at day 14 for H9 cell line. Scare bar, 100μm.
E. Percentage of the $GABA^+$ neurons at day 14 for H9 cell line. ***, P<0.001.
F. Percentage of the $GABA^+$ neurons (red) among the neurons ($TUJ1^+$ in the early stages from d17 to d21 for H9 cell line. n=3. *, p<0.05; **, p<0.01. *Error bars* represent S.E.
G. Percentage of the $GABA^+$ neurons (red) among the neurons ($TUJ1^+$) in the early stages from d17 to d21 for IMR90-4 cell line. n=3. *, p<0.05; **, p<0.01. *Error bars* represent S.E. Cell line, IMR90-4.
H. Percentage of the total cells expressing CR and CB during differentiation. More than 1,500 cells from random fields were manually counted in each condition. n=3. **, p<0.01. *Error bars* represent S.E. Cell line, IMR90-4.

Supplementary Tables

Supplementary Table S1. Gene ontology analysis of up-regulated and down-regulated genes in small molecule dosing group.

GO	Category	Description	Coun	%	Log10(P)	Log10(q)
GO:0030198	GO Biological	extracellular matrix organization	105	4.9388523	-28.48913353	-24.17776116
R-HSA-	Reactome Gene Sets	Cell Cycle	149	7.0084666	-26.3931634	-22.5589123
GO:0001568	GO Biological	blood vessel development	161	7.57290687	-24.25204385	-20.6396415
GO:0051301	GO Biological	cell division	132	6.2088429	-22.204316	-18.84718615
GO:0031589	GO Biological	cell-substrate adhesion	90	4.23330198	-21.29521135	-17.98383899
GO:0030155	GO Biological	regulation of cell adhesion	133	6.25587959	-18.66261352	-15.43042241
GO:0050767	GO Biological	regulation of neurogenesis	148	6.96142992	-18.55041453	-15.35298552
R-HSA-109582	Reactome Gene Sets	Hemostasis	128	6.02069614	-17.95458697	-14.81930586
GO:0050808	GO Biological	synapse organization	81	3.80997178	-17.81305654	-14.7321331
GO:0070848	GO Biological	response to growth factor	141	6.6321731	-17.69376111	-14.6558954
GO:0048729	GO Biological	tissue morphogenesis	124	5.83254939	-16.49727622	-13.51147481
GO:0048589	GO Biological	developmental growth	123	5.7855127	-15.58280232	-12.76279165
GO:0044772	GO Biological	mitotic cell cycle phase transition	107	5.03292568	-15.43410593	-12.62788354
GO:0007507	GO Biological	heart development	112	5.26810913	-14.7799645	-12.05965675
hsa05200	KEGG Pathway	Pathways in cancer	86	4.04515522	-13.72660259	-11.10542631
R-HSA-	Reactome Gene Sets	Non-integrin membrane-ECM	28	1.31702728	-13.64296346	-11.03916127
GO:0045596	GO Biological	negative regulation of cell	128	6.02069614	-13.3880433	-10.82485896
GO:0097435	GO Biological	supramolecular fiber organization	121	5.69143932	-13.31134658	-10.75584907
GO:0061448	GO Biological	connective tissue development	64	3.01034807	-13.15326209	-10.61274174
GO:0006260	GO Biological	DNA replication	65	3.05738476	-13.09920077	-10.5802201

"Count" is the number of genes in the user-provided lists with membership in the given ontology term. "%" is the percentage of all of the user-provided genes that are found in the given ontology term (only input genes with at least one ontology term annotation are included in the calculation). "Log10(P)" is the p-value in log base 10. "Log10(q)" is the multi-test adjusted p-value in log base 10.

Supplementary Table S2. GABAergic synapse pathways that were altered in small molecule dosing group by using KEGG pathway enrichment.

Category	Term	Kappa
**KEGG_PATHWAY**	hsa04727:GABAergic synapse	1
**KEGG_PATHWAY**	hsa04723:Retrograde endocannabinoid	0.715478882
**KEGG_PATHWAY**	hsa05032:Morphine addiction	0.697326898
**KEGG_PATHWAY**	hsa04725:Cholinergic synapse	0.644286883
**KEGG_PATHWAY**	hsa04713:Circadian entrainment	0.625579608
**GOTERM_BP_DIREC**	GO:0071377 cellular response to glucagon	0.60693479
**KEGG_PATHWAY**	hsa04726:Serotonergic synapse	0.603050647
**KEGG_PATHWAY**	hsa04724:Glutamatergic synapse	0.603050647
**KEGG_PATHWAY**	hsa04728:Dopaminergic synapse	0.525715844

Cohen's kappa coefficient (κ) is a statistic which measures inter-rater agreement for qualitative (categorical) items.

Supplementary Table S3. Primary Antibodies Used in This Study

Antibody	Isotype	Dilution	Source (cat. no.)
Calbindin (CB)	Rabbit IgG	1:1000	Abcam (AB1778)
Calretinin (CR)	Rabbit IgG	1:1000	Swant (7699/3H)
GABA	Rabbit IgG	1:1000	Sigma-Aldrich (A2052)
Map2	Mouse IgG	1:1000	Sigma-Aldrich (M1406)
Nkx2.1	Mouse IgG	1:500	Chemicon and Millipore (MAB5460)
Somatostatin and Receptor (SST)	Rat IgG	1:500	Chemicon and Millipore (MAB354)
β-III Tubulin	Mouse IgG	1:2000	Sigma-Aldrich (T8660)

Supplementary Table S4. Cell culture factors

Product	Company	Catalogue number
E8 (Essential 8)	Gibco	A1517001
DMEM/F-12	Gibco	11330
NEAA (MEM non-essential amino acids solution)	Gibco	11140
N2 SUPPLEMENT	Gibco	17502-048
VTN-NC (Vitronectin)	Gibco	A14700
FBS (Fetal Bovine Serum)	Gibco	10099-141
SAG	Millipore	566660
Forskolin	Tocris	1099
purmorphamine	Stemgent	04-0009
Trizol	ThermoFisher Scientific	15596018
Dispase	ThermoFisher Scientific	17105-041
PBS tablets	Medicago	09-9400-100
TrypLE	ThermoFisher Scientific	12604021
B27 Supplement	ThermoFisher Scientific	12587010

Supplementary Table S5. Cell culture medium

Reagent	Ingredient (Concentration)	Working Feature
E8 (Essential 8) Gibco, cat.no.A1517001	DMEM-F12<br>L-Ascorbic Acid Selenium<br>Holo-transferrin<br>Sodium butyrate<br>Insulin<br>bFGF<br>TGFß1	xeno-free,serum free
NIM (Nerual induced medium)	DMEM/F-12 (1X)<br>NEAA (100X)<br>N2 SUPPLEMENT (100X)	xeno-free,serum free
DMEM/F-12 Gibco, cat. no. 11330		xeno-free,serum free
NEAA (MEM non-essential amino acids solution) Gibco, cat. no. 11140		xeno-free,serum free
N2 SUPPLEMENT Gibco, cat. no. 17502-048	Human Transferrin (Holo)<br>Insulin Recombinant Full Chain<br>Progesterone<br>Putrescine<br>Selenite	Protein (human source)<br>Compound
VTN-NC (Vitronectin) Gibco, cat. no. A14700	5μg/ml	Human recombination

Supplementary Table S6. Quantitative PCR Primers Used in this Study

Target	Forward	Reverse
GAPDH	TCGACAGTCAGCCGCATCTTCTTT	ACCAAATCCGTTGACTCCGACCTT
Islet1	GTTTGAAATGTGCGGAGTGTAAT	TTCTTGCTGAAGCCGATGC
Lhx6	ACAGATCTACGCCAGCGACT	CATGGTGTCGTAGTGGATGC
Lhx8	CCAAAACCAGCAAAAAGAGC	TGGCGTGCTCTACAATTCTG
Nkx2.1	CGCATCCAATCTCAAGGAAT	CAGAGTGTGCCCAGAGTGAA
Pax6	ACAGATCTACGCCAGCGACT	CATGGTGTCGTAGTGGATGC

Supplemental Information

Materials and Methods

hPSC culture and neural differentiation in a feeder free system

hESCs (H9, passages 42-48, WiCell Agreement NO.16-W0060) and iPSCs (IMR90-4, passages 53–58, CCL-186) were maintained under feeder-free conditions coated with vitronectin (Life technology). For hPSCs passages, after 5-7 days of maintaining with daily changed E8 medium (Life technology), cells were dissociated into small pieces by using EDTA (Lonza, 1 mL /well) for 1-2 minutes, and reseeded at the density of 1×10^5 cells per well of a 6-well plate. For neural differentiation, hPSCs were detached by 1 U/ml dispase (Gibco) to form embryoid bodies (EBs), and then cultured in suspension in neural induction medium (NIM). After floating culture for 6-7 days, EBs were attached on 6-well plate or 3.5 cm^2 dishes, and then adherent cultured lasted for 7-10 days with NIM changed every other day. During the stage, the neural tube-like rosettes structure appeared and then the differentiating colonies became loosen and easy to be lifted. The rosette-containing colonies are detached and grown in suspension to form neuroepithelial spheres (NS). During the ventral differentiation, small molecules or sonic hedgehog would be used to pattern the neuroepithelial toward GABA progenitors. The floated neurospheres were then dissociated to single cell by TrypLE (Life technology) and plated at 20,000 cells/cm^2 on matrigel (BD Biosciences) and poly-l-ornithine (Sigma) pre-coated coverslips for further neuronal differentiation. The cell culture factors and reagents were listed in Supplementary Tables S4 and S5.

Microarray data

Microarray datasets GSE83896 was downloaded from the Gene Expression Omnibus database (GEO DataSets). GSM2221129, GSM2221130, and GSM2221131 were selected as control groups. GSM2221150, GSM2221151, and GSM2221152 were selected as experimental groups. A total of 6 samples were collected from the datasets. To obtain differentially expressed genes (DEGs) between control and experiment samples, the samples were divided into two groups. After GEO2R (http://www.ncbi.nlm.nih.gov/geo/geo2r/) analysis, results including adjusted P values (adj. P. Val) and log FC were provided. Cut-off criterion was set as adj. P.Val <0.01 and |log FC| >1. A list of candidate DEGs was obtained via the above methods. Gene ontology (GO) analysis and Kyoto Encyclopedia of Genes and Genomes (KEGG) pathway analysis are both integrated in the Database for Annotation, Visualization and Integrated Discovery (DAVID, http://david.abcc.ncifcrf.gov/) program. In this experiment, DEGs were uploaded onto DAVID in order to perform related GO and KEGG pathway enrichment analyses. The cut-off criterion was set as $P<0.05$. We first identified all statistically enriched terms, accumulative hypergeometric p-values and enrichment factors were calculated and used for filtering. We then selected a subset of representative terms from this cluster and convert them into a network layout. More specifically, each term is represented by a circle node, where its size is proportional to the number of input genes fall into that term, and its color represent its cluster identity. Terms with a similarity score > 0.3 are linked by an edge (the thickness of the edge represents the similarity score). The network is visualized with Cytoscape (v3.1.2) with "force-directed" layout and with edge bundled for clarity. One term from each cluster is selected to

have its term description shown as label.

Quantitative real-time polymerase chain reaction (qPCR)

When the neuroepithelial spheres were formed, cells were collected and dissociated with TRIzol reagent (Life Technologies). The RNA was extracted from neurospheres and diluted to 1µg with DAPC-treated water. According to the manufacturer's manual, SuperScript III First-Strand system (Life Technologies) was used to synthesize the cDNA. The qPCR was performed in a 20-µl reaction system, which included 4 µl cDNA, 2 µl random primers, 2 µl ddH$_2$O and 10 µl 2X SYBR Green RCR Master Mix (Roche). Glyceraldehyde-3-phosphate dehydrogenase (GAPDH) was used as a housekeeping gene. The primers used are listed in Supplementary Table S6.

Immunostaining for GINs

Cultured on coverslips were fixed in cold fresh 4% paraformaldehyde for 30 min and rinsed three times with phosphate buffered saline. Cells were treated with 0.2% TritonX-100 for 10 minutes and blocked in 10% donkey serum for 1 hour (brain slices: 0.5% TritonX-100 and 5% donkey serum for 1 hour). Cells were incubated at 4 °C overnight in primary antibody diluted with 0.1% triton and 5% donkey serum. On the second day, cells were incubated in secondary antibody diluted in 5% donkey serum for 30 min at room temperature. Coverslips were mounted for fluorescent imaging solution. The primary and secondary antibodies were listed in Supplementary Table S3.

Quantification of fluorescent images

The quantification of fluorescent images was analyzed using Image J. At least 9 random fields were selected and more than 1,500 cells of each cell line were counted, with more than 3 independent biological replicates performed. The number of nuclei labeled by Hoechst on each field was referred to as total cell numbers. The differences between purmorphamine group and small molecules group were tested by one-way ANOVA. A p value of <0.05 was considered to be statistically significant. All graphical data were presented as mean ± SEM.

Statistical analysis

SPSS software (version 20.0, SPSS, Inc., Chicago, IL, USA) was used for statistical analysis. Data are presented as a mean ± SEM. The comparison between two groups was performed by using the *t*-test. A p value of <0.05 was considered to be statistically significant. Analysis between groups were one-way ANOVA and two-way ANOVA.
